# Mesenchymal stem cells repair germinal cells of seminiferous tubules of sterile rats

**Published:** 2013-07

**Authors:** Malihezaman Monsefi, Bentolhoda Fereydouni, Leili Rohani, Tahereh Talaei

**Affiliations:** 1 *Department of Biology, College of Sciences, Shiraz University, Shiraz, Iran.*; 2 *Department of Anatomy, Laboratory for Stem Cell Research, School of Medicine, Shiraz University of Medical Sciences, Shiraz, Iran.*

**Keywords:** *Cell therapy*, *Immunohistochemistry*, *Germinal cells*, *Mesenchymal stem cells*

## Abstract

**Background: **Mesenchymal stem cells (MSCs) are undifferentiated cells that can differentiate and divide to other cell types. Transplantation of these cells to the different organs is used for curing various diseases.

**Objective:** The aim of this research was whether MSCs transplantation could treat the sterile testes.

**Materials and Methods:** In this experimental study, Donor MSCs were isolated from bone marrow of Wistar rats. The recipients were received 40 mg/kg of busulfan to stop endogenous spermatogenesis. The MSCs were injected into the left testes. Cell tracing was done by labeling the MSCs by 5-Bromo-2- Deoxy Uridine (BrdU). The immunohistochemical and morphometrical studies were performed to analysis the curing criteria.

**Results:** The number of spermatogonia (25.38±1.57), primary spermatocytes (55.41±1.62) and spermatozoids (4.95±1.30)×10^6^ in busulfan treated animals were decreased significantly as compared to the control group (33.35±1.78, 64.44±2.00) and (10.50±1.82)×10^6^ respectively but stem cells therapy help the spermatogenesis begin more effective in these animals (32.78±1.99, 63.59±2.01) and (9.81±1.33)×10^6^ respectively than the control group. The injected BrdU labeled mesenchymal stem cells differentiated to spermatogonia and spermatozoa in the seminiferous tubules of the infertile testis and also to the interstitial cells between tubules.

**Conclusion:** We concluded that testis of host infertile rats accepted transplanted MSCs. The transplanted MSCs could differentiate into germinal cells in testicular seminiferous tubules.

This article extracted from M.Sc. Thesis. (Bentolhoda Fereydouni)

## Introduction

Spermatogenesis is a process that occurs in the adult male testicular seminiferous tubules. Seminiferous tubules contain stem cells that proliferate and differentiate to spermatogenesis lineages. Also morphological changes of mammalian nucleoli was reported during spermatogenesis (1, 2). Infertility in men is most often caused by problems producing too few sperms or none at all or making abnormal sperm that prevent it from moving correctly to reach the egg and fertilize it. Busulfan, as alkylating agent, is used in chemotherapy. 

It adversely affects spermatogenesis in mammals and caused sterility, azoospermia, and testicular atrophy. Therefore, patients that have to use such drugs for cancer treatments, generally suffer from side effects such as infertility. Adult stem cells from bone marrow, referred to as mesenchymal stem cells or marrow stromal cells (MSCs), are defined as pluripotent cells and have the ability to differentiate into multiple mesodermal cells. According to the International Society for Cellular Therapy (ISCT), MSCs must express CD105, CD73 and CD90, and lack expression of CD45, CD34, CD14 or CD11b, CD79a or CD19 and HLA-DR surface molecules (3). 

MSCs can differentiate to mesodermal and non-mesodermal cells therefore; they are the best choice for therapeutic purposes (4, 5). Because of unique features of MSCs, their transplantation can improve various diseases. Also, injected MSCs into severe osteoarthritis of knee joints in goat showed the regeneration of the surgically amputated meniscus (6). In rodent stroke models one week after interrupting blood flow to the brain, MSCs injection results in the recovery of coordinate function (7, 8). MSCs secrete large quantities of bioactive factors that are both immunomodulatory and trophic. The trophic activity stimulates mitosis of tissue intrinsic progenitor cells (9). Regards to repairing potential of MSCs, we suggested that MSCs can improve germinal epithelial repairing potential in testicular seminiferous tubules. Therefore, this study was designed to investigate whether cell therapy with injection of MSCs could promote fertility potential in sterile male rats.

## Materials and methods

**Animals**

Wistar male rats weighting 210±50g were purchased from Razi Institute (Shiraz, Iran). The animals were adapted to the laboratory for two weeks prior to beginning of the experiments. The animals were maintained at controlled temperature (22-24^o^C) and a period of 12h lightness (6.00-18.00), and 12h darkness. Rats had free access to food and tap water. The animal experiments were approved by the Institutional Animal Ethics and Health Committee of the Biology Department of Shiraz University.

**Experimental design**

Male rats were sterilized with single dose IP injection of 40 mg/Kg busulfan (10). Busulfan was solved in 250 μL DMSO (dimethyl sulfoxide; Sigma, USA) and 250 μL distilled water (1:1) freshly. Animals were divided into 6 groups consist of: 1) rats that were received single dose of busulfan for sterility checking; 2) rats that received DMSO as single IP dose; 3) rats that were treated with labeled MSCs with BrdU (5-Bromo-2- Deoxy Uridine; Sigma, USA); 4) rats that were treated with culture medium 5) rats that were injected BrdU by IP as positive control for immunohistochemical staining; and finally 6) control group rats that were not received any treatment. 

**Histological studies**

After 2 months of busulfan or DMSO injection, left testis of control, busulfan, DMSO and MSC administered groups were removed and fixed in 4% buffered formalin solution for 1 week then the paraffin blocks were prepared (11). The blocks were sectioned at 6µm thickness and were stained with hematoxylin and eosin. For histomorphometrical studies, 10 circular random selected seminiferous tubules diameters, germinal epithelium diameters, spermatogonia and primary spermatocytes diameters and numbers were measured in each section (5 microscopic slides for each animal) using ocular micrometer (Zeiss, Germany) (12). 

**Sperm count and motility**

At the time of dissection, 1 cm of distal end of left vas deferens of control, busulfan DMSO and MSC treated groups were removed and put in 3 mL of Hank’s balance salt solution (HBSS) at 37^o^C. Sperms were collected by diffusion method (13). Sperm count was carried out after 10 min using a hemocytometer (14). Total sperm count was calculated using below formula: A=B×C×D where A is the total number of sperm per 1 mL of semen, B is the total number of sperm calculated per 0.1 mm^3^ of solution, C is the depth factor and D is the dilution factor (=3 mL).

**Isolation of MSCs**

MSCs were collected by flushing the femur and tibia of 6-8 weeks old Wistar male rats with culture media. Cells were plated in DMEM (Dulbeco’s Modified Eagle Medium; Gibco, UK) supplemented with 1% L. Glutamin (Gibco, UK), 1% penicillin/ streptomycin (Gibco, UK), and 15% Fetal Bovine Serum (Gibco, UK). Non-adherent cells were eliminated by a half medium change at day 3 and the whole medium was replaced weekly with fresh medium. The cells were grown for 2 weeks until almost confluency. The MSCs were subcultured up to 3^rd^ passages. 

**Flow cytometry**

The harvested cells were fixed with paraformaldehyde. The cells were washed and the non-specific binding sites were blocked by phosphate buffer saline (PBS) containing gout serum. The monoclonal FITC conjugated anti-rat THY-1 (CD90) and (CD34) antibodies (Sigma, USA) were added and incubated at 4^o^C for an hour. The cells were centrifuged in cold PBS at 2100 rpm for 5 min. The supernatant was removed and the cells were measured in the FL1 channel of flow cytometer (BD Company, USA). The percentages of the cells that reacted to CD90 and CD34 were analyzed by histogram using the WINmdi 2.9 software. 

**Transplantation of MSCs**

For tracing of MSCs in testes, they were labeled by adding 1 mmol BrdU (Sigma, USA) for 24 h to the cultures. The busulfan treated animals were anesthetized. 1.75×10^5^ MSCs were injected into the left testis. 8 weeks after transplantation, testes were removed and examined immunohistochemically for tracing of transplanted Brdu labeled MSCs.

**Immunohistochemical staining**

Rats were perfused by 10% buffered formalin under anesthesia. Then the longitudinal sections of testis prepared histologically. The specimens were mounted on poly-L lysine treated slides. Endogenous peroxidase was blocked by incubating the specimens in 1% H_2_O_2_ in PBS. To decondensing the nucleus, the sections were incubated in 2 NHCl for 30 min in 37^o^C. Then, retrial was performed with 0.1% trypsine for 30 min in 37^o^C. After washing with PBS, the non-specific binding site was masked by incubating the sections with goat serum for 15 min. The sections were incubated in anti-BrdU antibody (sigma, USA) in concentration of 1:70 in 4^o^C for overnight. 

The primary antibody dilution solution was PBS containing 1% BSA (Bovine Serum Albumin), 0.05% Tween 20 and 0.1% NaN_3_. The sections were then incubated with peroxidase conjugated anti-mouse secondary antibody (Sigma, USA) for 2hr at room temperature. Secondary antibody was diluted 1:100. Following final washing with PBS, the binding sites were visualized by incubating the sections in 0.03% diaminobenzidine (DAB; Sigma, USA) containing 200 µL H_2_O_2_ in PBS for 10 min. 

Then, the sections were counterstained with alcian blue (0.5%). The photographs were taken by digital camera. The BrdU positive sites were visualized as dark brown area. 

**Statistical analysis**

The data were analyzed using one-way ANOVA, followed by Tukey and Scheffe tests. Statistical analyses were performed using SPSS 11.5 software. P<0.05 was considered as a significant level. 

## Results

Histological examinations of testes in busulfan treatment group after two months revealed some degenerative changes such as seminiferous tubular atrophy and germinal epitheliums degenerations in the most of tubules. Sperm clusters and spermatids numbers were decreased in some tubules, spermatogonia and primary spermatocytes showed normal size but their numbers were also decreased significantly (Tables I, II). 

Some germinal cells showed pyknotic nuclei. The large vacuolated lumen occupied seminiferous tubules and the atrophic germinal epithelium covered peripheral zone of seminiferous tubules as thin band. Morphometrical measurements indicated the number of spermatogonia and primary spermatocytes decreased significantly compared to control group (p=0.02) but secondary spermatocytes and spermatids was absence in most of the seminiferous tubules. The seminiferous tubules diameter and germinal epithelium diameters also decreased significantly (p=0.01) compared to the control group (Table I). 

**Figure 1:**
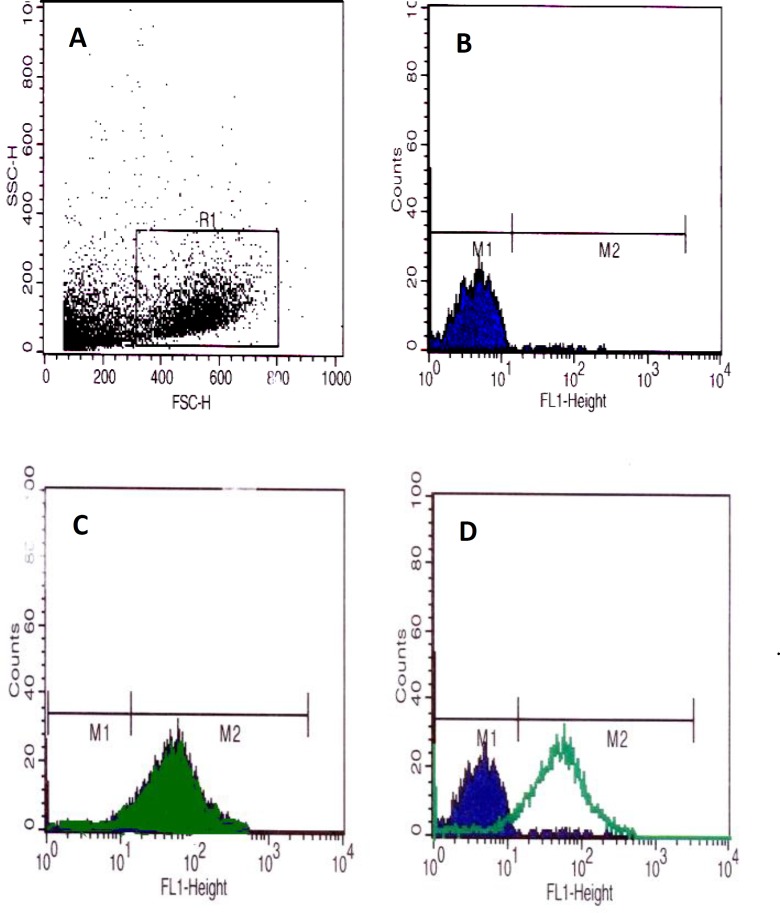
Flow cytometry histograms revealed that the separated cells from bone marrow of male rats reacted with antibody against CD90 markers. Dot blots in R1 square represented the normal MSCs population (A); dark blue carve in M1 zone were negative control for CD90 marker (B), adjustment of M1 and non mesenchymal stem cells population in A were noted. The green carve showed the CD90 labeling of MSCs (C); finally D histogram represented adjustment of two populations of CD90 labeling MSCs in M2 zone and non labeling cells in M1 zone. M2=91.5%.

**Figure 2:**
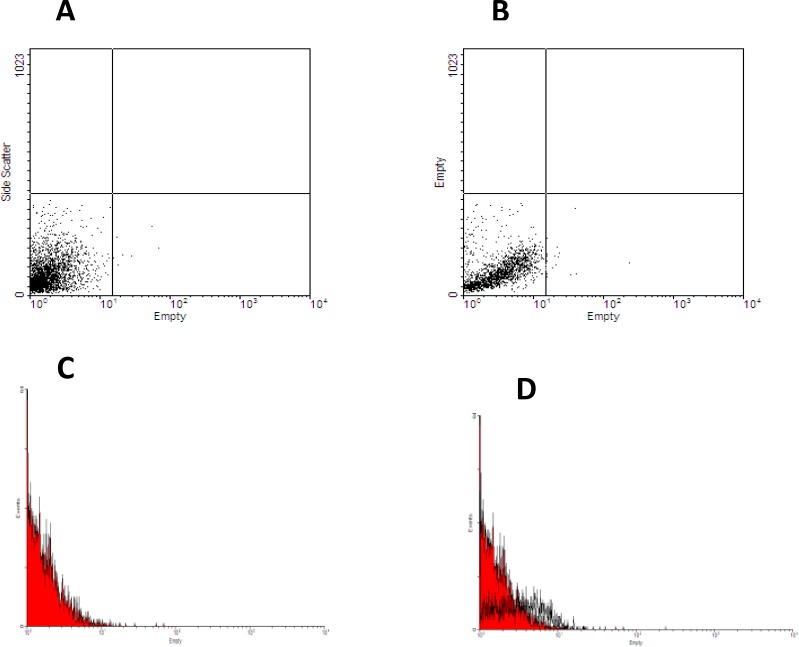
Flow cytometry histograms revealed that some separated cells from bone marrow of male rats were hematopoeitic stem cells and reacted with antibody against CD34 markers. Dot blots in (A) histogram represented the negative control for CD34 marker; Dot blots in (B) histogram represented hematopoeitic stem cells population separated from bone marrow of male rats; red carve (C) showed negative control for CD34 marker, finally D histogram represented adjustment of two populations of negative and positive CD34 labeling cells.

Significant decrease in sperms count in busulfan treated group was also observed (p=0.014) compared to the control group (Table II). There were no pathologic changes in structure and histomorphometrical examinations of testes that pre-exposed with DMSO (Tables I, II). Therefore, we concluded busulfan could destroy germinal cells of testicular seminiferous tubules and induce infertility in male rats. Mesenchymal stem cells subcultured up to third passages. These cells (91.5%) represented CD90 on their surface after flow cytometry analysis (Figure 1, A-D). Also they did not show any reaction for CD34 that is a specific marker of hematopoietic stem cells (Figure 2, A-D). Eight weeks after BrdU labeled MSCs injection to rats in busulfan treated group, some labeled cells were observed in the seminiferous tubules and in the interstitium of the testis. 

As figure 5 C was depicted, the labeled cells had the morphology like spermatogonia, spermatocytes, and spermatid, sperm and Leydig cells while these cells are absence in the control and medium injected groups. Seminiferous tubules of testes in the group that treated with labeled MSCs indicated some spermatogonia, primary spermatocytes, spermatids and sperms that reacted to immunohistochemical method (Figure 5, A-D). Therefore, injected MSCs differentiated to testicular germinal cells in their new niche. Also, MSCs differentiated to Leydig like cells that situated between testicular seminiferous tubules (Figure 6-E). Morphometrical studies revealed the seminiferous tubules diameters and their cells number and counting of MSC injected group had no significant differences compared to the control group (Tables I, II). Therefore, cell therapy could help the fast repair of pathological changes in testicular seminiferous tubules.

**Table I:** Histomorphometrical data of rat’s testes in busulfan, DMSO and MSC treatment groups compared to the control group

**Groups**	**Seminiferous tubules diameters (mm)**	**Germinal epithelium diameters (mm)**	**Luminal diameter** **(mm)**	**Spermatogonia diameter (μm)**	**Primary spermatocyte diameter (μm)**
Control	0.28 ± 0.03	0.27 ± 0.02	0.10 ± 0.01	6.19 ± 0.89	13.68 ± 1.80
Busulfan treatment	0.17 ± 0.03 *	0.18 ± 0.02 *	0.13 ± 0.02 *	6.24 ± 0.90	13.32 ± 1.85
DMSO treatment	0.26 ± 0.02	0.28 ± 0.01	0.12 ± 0.10	6.17 ± 0.81	13.56 ± 1.81
MSC injected group	0.27 ± 0.04	0.26 ± 0.03	0.12 ± 0.04	6.00 ± 0.79	13.54 ± 1.67

Data are shown as the mean±SD.

The data were analyzed using one-way ANOVA, followed by Tukey and Scheffe tests.

Seminiferous tubules diameters of busulfan treated compared to control, p=0.01. Germinal epithelium diameters of busulfan treated compared to control, p=0.01. Luminal diameter of busulfan treated compared to control, p=0.01

* significant difference with the control group (p<0.05).

**Table II:** Morphometrical measurements of spermatogenic cells in busulfan, DMSO and MSC treatment groups compared to the control group

**Groups**	**Spermatogonia numbers**	**Primary spermatocytes numbers**	**Sperm counts**
Control	33.35 ± 1.78	64.44 ± 2.00	(10.50 ± 1.82) ×10^6^
Busulfan treatment	25.38 ± 1.57 *	55.41 ± 1.62 *	(4.95 ± 1.30) ×10^6^ *
DMSO treatment	33.60 ± 1.71	64.69 ± 1.96	(11.01 ± 2.63) ×10^6^
MSC injected group	32.78 ± 1.99	63.59 ± 2.01	(9.81 ± 1.33) × 10^6^

Data are shown as the mean±SD.

The data were analyzed using one-way ANOVA, followed by Tukey and Scheffe tests.

Spermatogonia numbers of busulfan treated compared to control, p=0.02. Primary spermatocytes numbers of busulfan treated compared to control, p=0.02. Sperm count of busulfan treated compared to control, p=0.014.

* significant difference with the control group (p<0.05).

**Figure 3:**
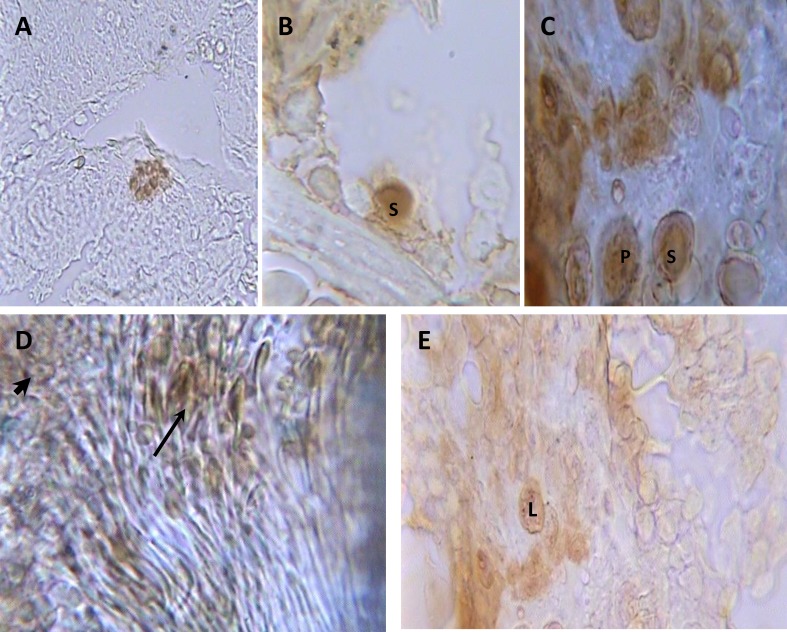
Photomicrograph of longitudinal sections of testes in BrdU labeled MSCs injected group after monoclonal anti BrdU staining and alcian blue counterstaining. Colony of MSCs (A) in seminiferous tubules (400×); spermatogonium (S) (B) and spermatogonia (S) and primary spermatocytes (P) (C), sperm cluster (thin arrow) and spermatids (thick arrow) (D) in seminiferous tubules (1000×) and (E) Leydig cells (L) between seminiferous tubules (1000×).

## Discussion

There are some techniques to improve the men infertility that has profound effects in the quality of their life. Infertility is a side effect of some drugs in men. Busulfan is used as chemotherapy agent for treatment of myeloid leukemia and before bone marrow transplantation. Treatment with such alkylating agents could induce apoptosis and destroyed mitotic cells such as spermatogonia in testicular seminiferous tubules and caused infertility (15). 

Cell therapy with adult stem cells is the new method for treatment of some disorders. Adult MSCs that isolated from bone marrow are able to differentiate into different mesodermal cell lineages including bone, cartilage, muscle, fat and other connective tissues cells (16). MSCs could differentiate into cardiac myocytes that therapeutically are used in sites of cardiac ischemia caused by cardiac myocytes apoptotic (17, 18). It appears that MSCs did not differentiate into different cells types in above examples but rather, the MSCs exerted their influence by the secretion of massive amounts of growth factors and cytokines to affect a therapeutic outcome. These effects call ‘trophic’ activity. In these processes, MSCs secreted bioactive molecules. These components have different roles such as: inhibit apoptosis and fibrosis or scarring at sites of injury, limit the domain of damage, stimulate angiogenesis, and finally stimulate tissue-specific and tissue-intrinsic progenitors to proliferate. The infused MSCs also have immunomodulatory roles. They turn off T cells surveillance and chronic inflammatory processes (19).

Male germ cells (sperms) are derived from primordial germ cells (PGCs) that are set aside early in embryogenesis (20). They arrive the genital ridge and are enclosed by Sertoli cells and became gonocytes. They proliferate for a few days and then stopped in G0/G1 phase until birth. Within a few days after birth, these cells resume proliferation to initiate spermatogenesis then migrated to the basement membrane of seminiferous tubules and became undifferentiated type A spermatogonia, the spermatogonial stem cells (SSCs). SSCs divided and differentiated to produce spermatozoa (21). SSCs division and differentiation defects can cause azoospermia, oligospermia or testicular seminiferious tubules atrophy. There are some reports to show injecting of MSCs into the atrophic seminiferous tubules could improve infertility and provided functional data in support of stem cell self-renewal, and increase in the number of stem cell during regeneration upon transplantation (22-27). 

Bone Marrow Stem Cell-derived germ cells were transplanted in seminiferous tubules of busulfan-treated mice then testes were examined by after 8-12 months. Immunhistochemical methods revealed these cells expressed the known molecular markers of primordial germ cells (fragilis, stella, Rnf17, Mvh and Oct4) as well as molecular markers of SSCs and spermatogonia including Rbm, c-Kit, Tex 18, Stra 8, Piwil 2, Dazl, Hsp90a, b1-and a6- integrins (22). MSCs from enhanced green florescence protein (EGFP) transgenic rats injected into immature (3 weeks old) rats testes. 

Two to three weeks after transplantation, testes were examined histochemically for capacity to differentiate into steroidogenic cells. The results showed that MSCs represent a useful source of stem cells for producing steroidogenic cells (23). BrdU labeled spermatogonia from 2-4 days mice injected to busulfan-treated mice. After 2 months testes were examined by immunohistochemical staining of anti-BrdU antibody and c-kit gen (molecular markers of spermatogonia). The results indicated that spermatogonia were survived in busulfan-treated mice (24). 

Induced pluripotent stem cells (iPSCs) have the ability to differentiate directly into advanced germ cell lineages in vitro such as postmeiotic, spermatid-like cells without genetic manipulation but not into spermatogonia, haploid spermatocytes, or spermatids. In vivo study of these cells showed that they can differentiate to UTF1-, PLZF-, and CDH1-positive spermatogonia-like cells, HIWI- and HILI-positive spermatocyte-like cells (25). Human adult and fetal somatic cell-derived iPSC lines in a similar manner to human embryonic stem cells (hESC) can differentiate to primordial germ cells and entered meiosis (a functional marker of germ cell formation and differentiation). In the other hand they can differentiate to haploid cells with characteristic staining of acrosin for spermatid (26). It may provide a useful platform for the study of infertility defects. 

Our results indicated single dose of busulfan (40 mg/Kg) induced oligospermia and spermatogenesis disorder that confirmed with the previous studies (15, 27, 28). MSCs transplanted to their new niche of atrophic testicular seminiferous tubules of sterile male rats could survive and settled down in the seminiferous tubules and interestitium. SSCs that originated of MSCs, proliferated and produced other germ cells of primary spermatocyte, spermatids and spermatozoids in some seminiferous tubules of recipient’s rats. Also MSCs existed in interstitial connective tissue between the seminiferous tubules and their morphology was like leydig cells. 

The repair mechanism is not clear however, it may be due to either proliferation of alive SSCs by growth factor that produced by MSCs or direct differentiation of MSCs to spermatogonia. The blood-testis barrier may help the MSCs to preserve from immunologic responses (29). The MSCs did not reveal in all testicular seminiferous tubules. It may be due to normal repair of germinal epitheliums after busulfan injection. Also it may relate to inadequate number of MSCs implantation after injection. In summary, we conclude that testis of host infertile rats accepted transplanted MSCs. The transplanted MSCs could differentiate into all types of germinal cells in testicular seminiferous tubules. 
